# A Semi-Dissolving Microneedle Patch Incorporating TEMPO-Oxidized Bacterial Cellulose Nanofibers for Enhanced Transdermal Delivery

**DOI:** 10.3390/polym12091873

**Published:** 2020-08-20

**Authors:** Ji Eun Song, Seung-Hyun Jun, Sun-Gyoo Park, Nae-Gyu Kang

**Affiliations:** LG Household and Health Care R&D Center, Seoul 07795, Korea; sos6934@lghnh.com (J.E.S.); junsh@lghnh.com (S.-H.J.); skparke@lghnh.com (S.-G.P.)

**Keywords:** semi-dissolving microneedles, bacterial cellulose nanofibers, bio-based polymer microneedles, two-layer microneedles, enhanced transdermal delivery, drug inlet holes

## Abstract

Although dissolving microneedles have garnered considerable attention as transdermal delivery tools, insufficient drug loading remains a challenge owing to their small dimension. Herein, we report a one-step process of synthesizing semi-dissolving microneedle (SDMN) patches that enable effective transdermal drug delivery without loading drugs themselves by introducing TEMPO-oxidized bacterial cellulose nanofibers (TOBCNs), which are well dispersed, while retaining their unique properties in the aqueous phase. The SDMN patch fabricated by the micro-molding of a TOBCN/hydrophilic biopolymer mixture had a two-layer structure comprising a water-soluble needle layer and a TOBCN-containing insoluble backing layer. Moreover, the SDMN patch, which had a hole in the backing layer where TOBCNs are distributed uniformly, could offer novel advantages for the delivery of large quantities of active ingredients. In vitro permeation analysis confirmed that TOBCNs with high water absorption capacity could serve as drug reservoirs. Upon SDMN insertion and the application of drug aqueous solution through the drug inlet hole, the TOBCNs rapidly absorbed the solution and supplied it to the needle layer. Simultaneously, the needle layer dissolved in body fluids and the drug solution to form micro-channels, which enabled the delivery of larger quantities of drugs to the skin compared to that enabled by solution application alone.

## 1. Introduction 

The stratum corneum (SC), which is the outermost layer of the skin with a thickness of 10 to 15 μm, acts as a strong barrier to transdermal drug delivery systems. Microneedles that are a few hundred micrometers in length can physically pierce the skin with minimal invasion, which enables the simple, painless, and effective delivery of active ingredients into the skin. Their applicability has been widely studied in various fields, such as cosmetics, biomedicines, and vaccines [1,2,3,4,5]. Beginning with a study [6] in which microneedles consisting of silicon were observed to increase the skin permeability of calcein, various solid microneedles (SMNs) fabricated from metal, ceramic, or glass have been reported [7,8,9]. SMNs create transient micro-channels in the skin through which drugs that are applied topically or coated on the needles are delivered. However, if the needles break within the skin, they can remain within the skin for a long time and cause adverse events such as inflammatory responses.

Meanwhile, dissolving microneedles (DMNs), which are primarily composed of water-soluble polymers, are dissolved following skin penetration, and the drug encapsulated within the matrix is released. Compared to SMNs, DMNs offer various advantages, such as ease of production, convenience and safety of use. However, owing to the water-soluble nature of DMNs, the majority of active compounds that can be loaded in DMNs have been limited to hydrophilic materials. It is difficult to directly incorporate lipophilic drugs into the polymeric matrix of DMNs, as this requires additional processing techniques such as emulsification [10] or encapsulation [11,12]. In addition, the small dimension of microneedles limits the loading quantity of the active compound and might lead to insufficient efficacy. During the process of DMN patch fabrication, the active compounds are often degraded. Therefore, the challenge of overcoming the loading limitation of DMNs while preventing the degradation of biologically active compounds remains.

With respect to these issues, several studies have been conducted recently to improve the drug delivery potential of DMNs. The effects of the combined application of a DMN patch and a topical formulation, each containing the same active compound, have been investigated [13,14]. Combined approaches have led to improved transdermal delivery and efficacy compared to the individual applications of DMN or topical formulations. Another study reported the use of a two-phase (hydrophilic and lipophilic phase) delivery system consisting of a DMN patch (containing adenosine) and horse oil spread on the edge of the patch [15]. The oil phase was delivered into the skin through the channels created by the DMNs, and adenosine, a hydrophilic drug loaded within the DMNs, was delivered. Nevertheless, there is a persistent demand for the development of a novel DMN system to realize the delivery of a broad range of active compounds, while maintaining their bioavailability regardless of their drug-loading capacity.

Cellulose, a natural polymer produced by plants and microorganisms, is the most abundant biomass [16]. Currently, cellulose nanofibers are being investigated widely as promising tools for biomedical [17,18,19], cosmetic [20,21], and electrochemical applications [22] owing to their inherent biocompatibility, ease of chemical modification, and mechanical properties. In general, the type of cellulose used for DMNs is plant-derived carboxymethyl cellulose (CMC) which is used to maintain the physical properties and the shape of the object after the drying process [23]. However, CMC does not exhibit unique structural properties; it simply serves as a polymer.

Bacterial cellulose (BC) is a potential natural polymer produced by certain bacterial species such as *Acetobacter xylinum*. BC nanofibers offer various advantages compared to plant cellulose, including high water absorption and retention capacity, high mechanical strength, low solubility, resistance to degradation, and a uniform and nano-scaled fiber network structure [24,25,26,27,28,29]. Moreover, BC can be produced in a range of shapes and texture, such as filaments, films, spheres, multi-shaped pulps, particles, and whiskers among others. Although BC needs to be modified to make it water-dispersible for various applications, the requirement for these processes have been limited using conventional preparation methods for plant cellulose nanofibers. Based on the increasing demand for water-dispersible BC, we recently reported the production of water-dispersed bacterial cellulose nanofibers via TEMPO (2,2,6,6-tetramethyl-1-piperidine-*N*-oxy radical)-mediated oxidation, which converts the surface hydroxyl of cellulose into charged carboxyl entities [30,31]. In particular, TEMPO-oxidized bacterial cellulose nanofibers (TOBCNs) were observed to be highly dispersed whilst retaining their physical fiber structure in aqueous solution, and exhibited excellent water absorbance capacity [30]. The unique properties of TOBCNs could be utilized in the development of a novel DMN system that creates a two-layer microneedle without two-step casting, and also rapidly absorbs externally supplied drugs in aqueous solutions and delivers them sequentially into the skin though the microneedle channels.

**Figure 1:**
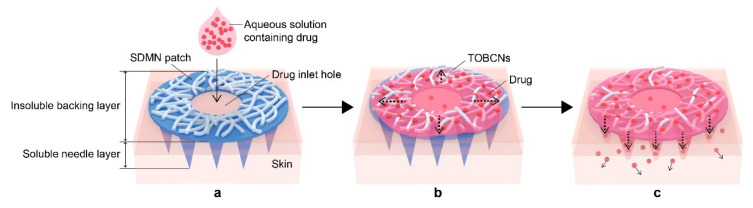
Step-wise illustration of the transdermal delivery of a drug loaded in topical aqueous solution by a semi-dissolving microneedle (SDMN) patch. (**a**) The SDMN patch, consisting of a soluble microneedle layer and an insoluble backing layer where TEMPO-oxidized bacterial cellulose nanofibers (TOBCNs) are evenly distributed, is applied to the skin. Sequentially, an aqueous drug solution is injected through the drug inlet hole in the backing layer of the patch. (**b**) The TOBCNs rapidly absorb the drug solution while the needle layer is dissolved, thereby creating micro-channels. (**c**) The TOBCNs serve as a drug reservoir that continuously delivers the drug to the skin via the micro-channels created by the dissolved needles.

Here, we designed a semi-dissolving microneedle (SDMN) patch by introducing TOBCNs with high water affinity along with water-insolubility to overcome the limitations of conventional DMNs; this enabled transdermal delivery without limiting the quantities or types of drugs. Although the SDMN patch was fabricated from a TOBCN/water-soluble biopolymer (hyaluronic acid (HA) and CMC) aqueous mixture using a conventional micro-molding technique, the TOBCNs were located only in the backing layer, which resulted in the formation of a two-layer SDMN patch. As shown in Figure 1, when a drug-containing aqueous solution is injected through the drug inlet hole in the backing layer of the patch, the solution spreads rapidly throughout the patch due to the presence of TOBCNs, which are distributed evenly in the backing layer. As the needle layer dissolves in body fluids and the aqueous drug solution simultaneously, the drug is readily delivered to the skin via the micro-channels created by the dissolved needles. The effects of transdermal drug delivery using the SDMN patch were visualized using model drugs, and in vitro skin permeation studies using fluorescein and nanocapsulated retinol were conducted as well. The SDMN patches facilitated enhanced transdermal delivery without drug-loading into the patches, and also enabled the delivery of a broad range of drugs while avoiding drug breakdown.

## 2. Materials and Methods

### 2.1. Materials

Hyaluronic acid (HA, 5 kDa) was purchased from SK bioland (Cheonan, Korea). Sodium carboxymethyl cellulose (CMC, 90 kDa), trypan blue solution (0.4%), Rhodamine B (≥95%) and fluorescein (95%) was purchased from Sigma-Aldrich (St. Louis, MO, USA). Trehalose was purchased from Hayashibara Co. Ltd. (Okayama, Japan). Glycerin was purchased from LG Household & Healthcare (Seoul, Korea). Aqua Cellulose Solution^TM^ (1.5% TOBCNs, 95.5% deionized water, and 3% hexandiol), which was prepared as we recently reported [31], was provided by The Garden of Natural Solution (Osan, Korea). Phosphate-buffered saline (PBS) was purchased from GIBCO (Grand Island, NY, USA). Retinol was purchased as a form of mixture with Tween 20 (1/1 by *w*/*w*) from BASF (Retinol 50C, Ludwigshafen, Germany). Palmester 3580 (caprylic/capric triacylglycerols) was purchased from KLK OLEO (Selangor, Malaysia). Compritol 888 ATO (glyceryl behenate) was purchased from Gattefossé (Saint-Priest, France). Stearylamine (>85.0%) was purchased from TCI (Tokyo, Japan). Polysorbate 60 (Tween 60) was purchased from Croda Inc. (Barcelona, Spain). Polydimethylsiloxane (PDMS, Sylgard 184) was purchased from Dow Corning (Midland, MI, USA).

Porcine cadaver skin (2.5 cm × 2.5 cm × 1 mm) was purchased from APURES Co. Ltd. (Pyeongtaek, Korea) for in vitro skin permeation study. SC layers were collected by tape stripping using D-Squame tapes and a D-Squame pressure applicator (CuDerm, Dallas, TX, USA).

### 2.2. Preparation of SDMN Patch

We used the micro-molding technique [32], which has been widely utilized to fabricate conventional DMN patch, to fabricate the SDMN patch. Microneedle molds were prepared by casting the PDMS precursor solution over a microneedle array master (LG Electronics, Pyeongtaek, Korea), which contains 1084 pyramidal-shaped microneedles (250 μm height, 125 μm base width, and 400 μm tip-to-tip spacing), followed by curing at 70 °C for 12 h. For the SDMN mixture solution, HA (0.22 g), CMC (0.18 g), trehalose (0.30 g), glycerin (0.23 g) and Aqua Cellulose Solution^TM^ (1.5 wt% TOBCNs, 5.0 g) were mixed with deionized water (4.07 g). The mixture was casted onto the PDMS negative mold, vacuumed for 30 min and dried at 50 °C for 3 h. Then, the SDMN circular patch (1.5 cm in diameter) was attached to a circular hydrocolloid adhesive with a circular hole (1 cm in diameter) in the middle for skin adhesion, then separated from the mold, and a circular hole (0.6 cm in diameter) for the drug inlet was punched at the center of the patch. For comparison, DMN patches without TOBCNs were also fabricated.

### 2.3. Morphological and Mechanical Characterization of SDMN Patch

The morphology of the microneedles was observed using an optical microscope (Leica DM 1000, Leica, Solms, Germany) and a scanning electron microscope (SEM, S-4800, HITACHI, Tokyo, Japan). To validate the skin penetration ability, the prepared SDMN patches were applied on the porcine skins with a force of 20 N for 10 s. After the removal of the SDMN patch, the microchannels created on the skin were selectively stained with trypan blue solution for 1 min, and excess solution was wiped off. The stained microchannels were observed using an optical microscope and compared to the number of needles applied.

The insertion force of the SDMN patch was analyzed by a TA.XT Plus Texture Analyzer (Stable Micro Systems, Surrey, UK). The fresh porcine skin was fixed on a polystyrene foam block that mimics soft tissue under the skin [33] and placed on a platform of the machine. The SDMN patch which was mounted on the cylindrical stainless-steel probe (2.5 cm in diameter) using double-sided adhesive tape (Scotch double-coated tape 665, 3M, St. Paul, MN, USA) was moved towards the porcine skin at a speed of 0.05 mm/s. The loading force and displacement were recorded until the loading force reached 30 N.

### 2.4. Skin Distribution Analysis of Model Drug

The distribution of model drugs loaded in topical aqueous solution was observed using rhodamine B and fluorescein. Firstly, the porcine skin, wiped off with a tissue, was placed on an aluminum foil. Then, a SDMN patch was attached to the skin with a force of 20 N for 10 s, and the skin with the SDMN was carefully mounted on the Franz diffusion cell having a 2.83 cm^2^ of diffusion area. The receptor solution was composed of 2.5 mL of PBS buffer (pH 7.4) to achieve sink condition. To confirm the horizontal diffusion of an aqueous drug solution, photographs were taken after 30 min of rhodamine B aqueous solution (300 μg/mL, 100 μL) application. For comparison, the skin with DMN without TOBCNs was used as control. Additionally, to assess the vertical diffusion, fluorescein aqueous solution (50 μg/mL, 2 mL) was applied onto the drug inlet hole in the backing layer of the SDMN patch, which was inserted in the porcine skin, and maintained at 37 °C and 50% relative humidity for 3.5 h. A control experiment was performed with the same fluorescein solution on the porcine skin without the SDMN patch. After the removal of the remaining fluorescein solution and the SDMN patch, the skin cross-sectional images were analyzed via a microscope (EVOS FL Auto 2, Thermo Fisher Scientific, Waltham, MA, USA) to verify vertical diffusion of fluorescein.

### 2.5. In Vitro Skin Permeation Studies

The in vitro permeation study was carried out with the Franz diffusion cell, as described above. The porcine skins and receptor mediums were withdrawn after 3.5 and 24 h of application. To determine the amount of sample absorbed in the SC, tape-stripping was repeated on the same skin area 3 times with a pressure applicator and stripping tapes. Each tape was collected in an individual 1.5 mL Eppendorf tube (Eppendorf, Hamburg, Germany), followed by adding 1 mL of PBS solution to the tube, and maintained for 24 h to sufficiently extract the fluorescein absorbed in the SC. The solutions for the three tapes were analyzed to determine the sample absorbed in SC. Meanwhile, the tape-stripped porcine skin was placed in plastic zipper bag, soaked in distilled water at 70 °C for 30 s, and separated into epidermis and dermis by pushing gently the skin surface with a pusher. Each tissue was homogenized in PBS solution (1 mL) using a Precellys 24 homogenizer (Bertin Technologies, Montigny, France), and centrifuged (Centrifuge 5427R, Eppendorf, Hamburg, Germany) at 13,000 rpm for 5 min. Each supernatant was collected to determine the permeation amount of fluorescein by using a fluorescence spectrometer (Varioskan Lux, Thermo Fisher Scientific, Waltham, MA, USA). The statistical tools within the Microsoft Excel program (Redmond, WA, USA) were used to calculate *p* values. The *p* values were calculated by a Student’s *t*-test with equal variance to compare the groups with and without SDMN patch application.

Prior to the in vitro permeation study for retinol, retinol was encapsulated with lipids as the capsule matrix. Briefly, retinol 50C (5 g) was dissolved in Palmester 3580 (2.5 g) and mixed with melted Compritol 888 ATO (3 g) and stearylamine (2 g). The lipid mixture was added dropwise to Tween 60 aqueous solution (10 wt%) at 80 °C under vigorous stirring, resulting in pre-emulsion. The pre-emulsion was further sonicated for 10 min and cooled down to room temperature and stored in a refrigerator before use. The hydrodynamic diameter of the retinol nanocapsules was measured by the dynamic light scattering (Nano-ZS90, Malvern Instruments, Worcestershire, UK). After the insertion of the SDMN patch into the porcine skin, the aqueous retinol nanocapsule dispersion (20.0 μg of retinol) was applied to the drug inlet hole of the SDMN patch and other processes were carried out as described above.

## 3. Results and Discussion

### 3.1. SDMN Preparation and Characterization

**Figure 2:**
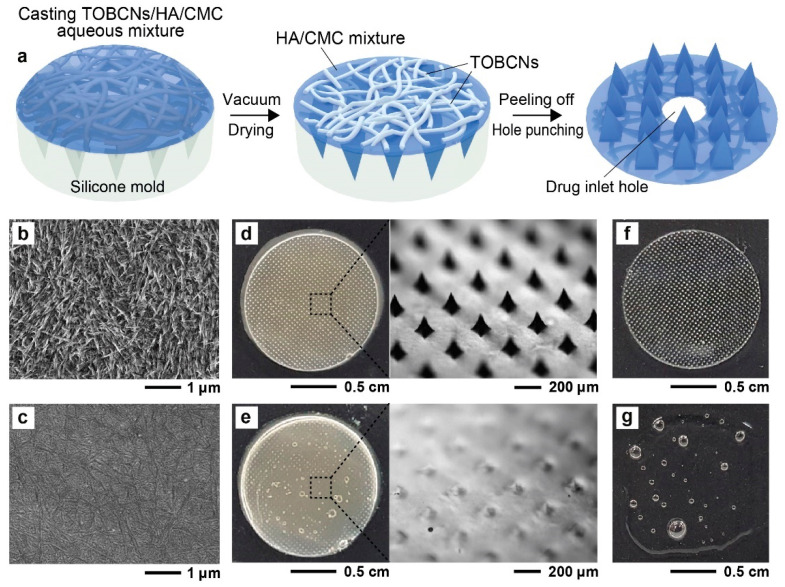
(**a**) Schematic illustration of the one-step fabrication of the SDMN patch. (**b**,**c**) SEM images depicting (**b**) the TOBCNs and (**c**) the backing layer of the SDMN patch. (**d**,**e**) Dissolution test for the SDMN patch (**d**) before and (**e**) after dropping water on the patch. Microscopic images on the right indicate the morphological changes in the SDMN patch (from the black dotted boxes in (**d**,**e**)). (**f**,**g**) Images of the DMN patches without TOBCNs (**f**) before and (**g**) after dropping water onto the patch. Abbreviations: HA, hyaluronic acid; CMC, carboxymethyl cellulose.

The SDMN patch was fabricated using the micro-molding method [32], which has been adopted previously to prepare conventional dissolving microneedles, as shown in Figure 2a (refer to Section 2). Briefly, an aqueous mixture, consisting of 50% Aqua Cellulose Solution^TM^ (containing 1.5% TOBCNs, Figure 2b), 2.2% HA, 1.8% CMC, 3.0% trehalose, 2.3% glycerin, and 40.7% deionized water, was cast on a negative silicone mold. Then, the aqueous mixture-loaded mold was vacuumed and dried at 50 °C for 3 h. After gently detaching the patch from the mold, a circular hole was punched into the backing layer of the patch for drug injection.

Nano-fibrous polymer microneedles with two-layer structures were developed earlier by the two-step casting of a silk fibroin solution and a prepolymer solution for fabricating the needle matrix [34,35]. In contrast, we found that the structural characteristics of TOBCNs enabled the one-step synthesis of two-layer microneedles in which TOBCNs are loaded only in a single layer. We observed that TOBCNs were localized only to the backing layer of the SDMN patch (Figure 2c), which exhibited a uniform distribution of TOBCNs. However, TOBCNs were rarely found on the needle surface and the front surface of the patch (Figure S1). Due to the small diameter (average diameter of 80 nm) and highly dispersed properties of TOBCNs, they exhibited three-dimensional (3D) and highly entangled network structures, which prevented the packaging of TOBCNs in the microneedle mold cavity. As a result, the TOBCN network structures were located only in the backing layer of the patch, which enabled the synthesis of the two-layer microneedles through single-step casting. To further confirm the dual structure of the SDMN patch, a water droplet (100 μL) was dropped on the SDMN patch (Figure 2d, left), and for comparison, the same procedure was performed using a conventional DMN patch without TOBCNs (Figure 2f). In contrast to the DMN patch, which completely dissolved in water (Figure 2g), the SDMN patch containing the water-insoluble TOBCNs in the backing layer retained its shape after the water droplet evaporated (Figure 2e, left). Meanwhile, the pyramid- shaped microneedle layer (Figure 2d, right) dissolved completely in the water droplet (Figure 2e, right), which indicates that the needle layer did not contain TOBCNs. These results clearly highlight the unique semi-dissolving characteristics of the SDMN patch, where the dissolving needle layer is composed of water-soluble compounds and the water-insoluble backing layer contains TOBCNs.

**Figure 3:**
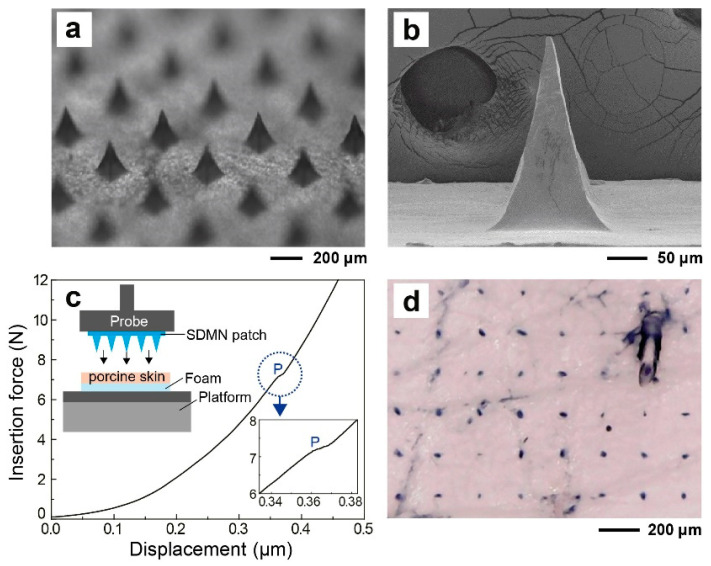
(**a**,**b**) Images showing the structure of the SDMN patch: (**a**) a low-magnification optical image and (**b**) a high-magnification SEM image. (**c**) A schematic of the experimental setup (inset) and a force–displacement curve of the SDMN during the insertion process of the SDMN into porcine skin. The force at the point “P” represents the penetration force to be required for porcine skin penetration. (**d**) To confirm the penetrating properties, the porcine skin was stained with trypan blue after the application of the SDMN patch. The blue dots on the skin indicate the micropores created by the microneedles.

The fabricated SDMN patches are shown in Figure 3a,b. Each patch consisted of 1084 microneedles that had a pyramidal shape with uniform dimensions. The height, base width, and tip-to-tip distance of the microneedles were 250 ± 7, 125 ± 3, and 400 ± 4 μm, respectively. The SDMNs should have adequate mechanical strength to penetrate the SC to deliver active compounds across the skin barrier. To investigate the skin penetration performance of the SDMN, the insertion force during the insertion process of the SDMN into porcine skin was measured (Figure 3c). The resistance force gradually increased with the displacement due to the inherent resistance property of skin after the SDMN contacted the skin. When the insertion force increased with the displacement reached the rupture limit of the porcine skin, the microneedles penetrated the skin at the point “P” shown in Figure 3c. The minimum force required for microneedles penetrating the SC is defined as the penetration force [36,37] which for the SDMN patch comprising 1084 microneedles was approximately 7.2 N. Since the penetration force of a large number of microneedles might be slightly different from each other, the penetration point (P) was certainly broad, rather than clearly observed. The average thumb force that an adult presses the microneedles with is approximately 20 N [38]. Therefore, the SDMN patch had adequate mechanical strength to penetrate the skin with thumb force application. In addition, using a different method, the skin penetration rate of the needle was determined to indirectly evaluate the strength of the needle. As shown in Figure 3d, micro-channels were created with a force of 20 N while attaching the SDMN patch to porcine skin and were dyed. As indicated by the ratio of the number of holes to those of needles, more than 90% of the needles had adequate strength to penetrate the skin.

### 3.2. Distribution Analysis of Model Drugs

**Figure 4:**
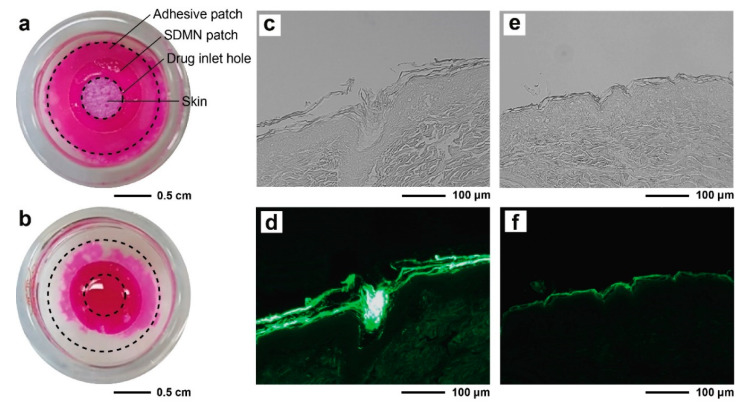
(**a**,**b**) Images depicting porcine skin with (**a**) an SDMN patch and (**b**) a dissolving microneedles (DMNs) patch without TOBCNs, mounted on Franz diffusion cells, followed by the application of rhodamine B aqueous solution into the drug inlet hole in the backing layer. The images were recorded after 30 min and the dotted circles indicate the microneedle patches. Cross-sectional (**c**,**e**) bright-field and (**d**,**f**) corresponding fluorescence images, after the application of fluorescein aqueous solution (**c**,**d**) in the presence of SDMN patches, and (**e**,**f**) in the absence of SDMN patches (solution-only treatment) for 3.5 h.

To date, several strategies that combine microneedles with drug reservoirs have been developed [39,40,41,42], such as liquid drug reservoir-integrated ceramic porous microneedles [39] or hydrogel-forming microneedle arrays combined with a drug-loaded adhesive backing layer [40,41]. However, in each of these approaches, it is necessary for the microneedles to remain inserted in the skin for a considerable period, which can cause discomfort to the patients and also increase the risk of infection or the chances of the retention of needle residue. In contrast, the SDMN patch, which consists of a dissolving needle layer that does not leave biohazardous sharp waste, and a backing layer containing TOBCNs as a drug reservoir, could provide a high storage capacity for an aqueous drug solution. Accordingly, we investigated whether the backing layer of the SDMN patch, which itself did not contain the drug, could rapidly absorb and retain an externally injected model drug aqueous solution, and simultaneously deliver it to the needle layer. The SDMN patch, the backing layer of which was attached to a hydrocolloid adhesive with a hole (1 cm in diameter) for skin adhesion, was applied to porcine skin, and an aqueous rhodamine B solution was used as a model drug and was subsequently injected into the drug inlet hole (0.6 cm in diameter) in the backing layer. The hole in the backing layer plays an important role in the immediate introduction of the aqueous drug solution between the backing and needle layers. As shown in Figure 4a, the rhodamine B aqueous solution was spread evenly over the backing layer region where the SDMN patch was attached. The drug solution was absorbed directly into the backing layer and did not flow outward, which would be convenient for the user. Even though the DMN patch without TOBCNs had the same hole structure as the SDMN patch, only a part of the injected drug solution was introduced and distributed non-uniformly on the backing layer, whereas the rest formed a droplet on the skin (Figure 4b). The results clearly demonstrate that the TOBCNs, which had high water absorption and retention capacity, were uniformly distributed in the patch backing layer, such that the aqueous drug solution could horizontally diffuse throughout the patch along the TOBCNs.

The vertical distribution of the drug in the skin after DMN patch application was also analyzed to confirm transdermal delivery through the patch. Figure 4c,d show the section of skin after the insertion of the SDMN patch, followed by the application of fluorescein aqueous solution for 3.5 h. Due to contact with body fluids and the injected drug solution, the needle layer was dissolved, which led to the formation of micro-channels in the skin that functioned as pathways for rapid drug delivery. When compared to the image of the application of the drug solution alone (Figure 4e,f), the strong fluorescent green color of fluorescein, which was deeply distributed in the skin, was observed in this image. Meanwhile, when the fluorescein solution was applied without the attachment of the SDMN patch, weak fluorescence was observed only in the SC layer. Taken together, these results indicate that the SDMN patches were able to deliver drugs horizontally and vertically, without taking up the drugs themselves.

### 3.3. In Vitro Skin Permeation Studies

**Figure 5:**
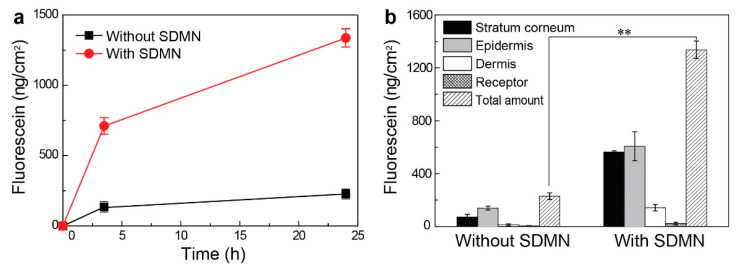
(**a**) In vitro porcine skin permeation profile of fluorescein using aqueous solution only and using SDMN-combined application. (**b**) Compartmental distribution of fluorescein in the stratum corneum (SC), epidermis, dermis and receptor after 24 h of application in the absence and presence of the SDMN patch. Mean ± SD, *n* = 2. ** *p* < 0.01, statistically significant difference between the two groups (without and with SDMN).

The effect of the SDMN patches on the skin permeation of active compounds was quantitatively evaluated using the Franz cell technique. In the in vitro permeation studies, the SDMN patch was attached to porcine skin composed of SC, epidermis, and upper dermis, and was then carefully mounted on a Franz diffusion cell; this represented the SDMN-combined group. Subsequently, fluorescein aqueous solution was used as a model drug, and its permeation over time was evaluated (Figure 5a). In a comparative study, only aqueous fluorescein solution was applied without SDMN patch application; this represented the control group.

After 3.5 h of application, 711 ± 57 ng/cm^2^ of fluorescein was delivered into the skin and the receptor in the SDMN-combined group, while 135 ± 4 ng/cm^2^ of fluorescein was delivered in the control group (solution-only treatment). Furthermore, after 24 h of application, the SDMN-combined group showed 1336 ± 66 ng/cm^2^ of fluorescein delivery, which was 5.8 times higher than that in the control group (230 ± 24 ng/cm^2^). Although the same concentration and quantity of fluorescein aqueous solution were used, the permeation rates differed significantly between the two groups; the initial permeation rate of the SDMN group for 3.5 h was significantly greater than that of the control group. In other words, passive diffusion through the skin at a relatively slow rate occurred in the control group, whereas the SC was physically penetrated in the SDMN group, which ensured the rapid delivery of active ingredients into deep layers of the skin. This indicated that the SDMN patch could successfully deliver fluorescein even though the microneedle patch did not contain the compound.

We further analyzed the relative distributions of fluorescein in the porcine skins from the two groups after 24 h of application (Figure 5b). In the control group, there were traces of fluorescein in the dermis (13.1 ± 7.4 ng/cm^2^) and receptor (4.6 ± 2.8 ng/cm^2^), and no significant change was observed compared to the values at 3.5 h (9.5 ± 4.7 and 0.3 ± 0.2 ng/cm^2^ for the dermis and receptor, respectively; refer to Figure S2). In contrast, the SDMN-combined group showed a further increase in the quantity of fluorescein that permeated into the dermis and receptor (from 34.5 ± 19.8 and 2.8 ± 0.4 ng/cm^2^ at 3.5 h to 142.2 ± 24.6 and 23.4 ± 9.8 ng/cm^2^ at 24 h for the dermis and receptor, respectively). Although the majority of permeated fluorescein was present in the SC and epidermis (88% of total permeated fluorescein), due to the relatively short height of the microneedle (~250 μm) compared to the skin thickness, the results clearly indicate that as a skin penetration enhancer, the SDMN patch could effectively deliver large quantities of relatively small water-soluble molecules.

To further demonstrate the versatility of the SDMN patches, we attempted to deliver retinol nanocapsules, which are significantly larger than fluorescein. Retinol, a lipophilic vitamin, promotes epidermal cell proliferation, which reduces skin wrinkling and an uneven tone [43]. However, since retinol is chemically unstable and readily oxidized, several retinol encapsulation methods have been developed with an aim to improve its stability [44,45,46,47]. Moreover, DMN patches are primarily composed of hydrophilic polymers and require a high-temperature drying process, which further complicates the process of the homogeneous loading of lipophilic retinol while maintaining its bioavailability. Even if retinol is loaded in an encapsulated form, its efficacy may not be ensured due to the limited loading efficiency of the capsules and DMNs, respectively [11]. Herein, we demonstrated that the SDMN patch could effectively deliver a sufficient level of nanocapsulated retinol even when retinol was not contained in the needles.

**Figure 6:**
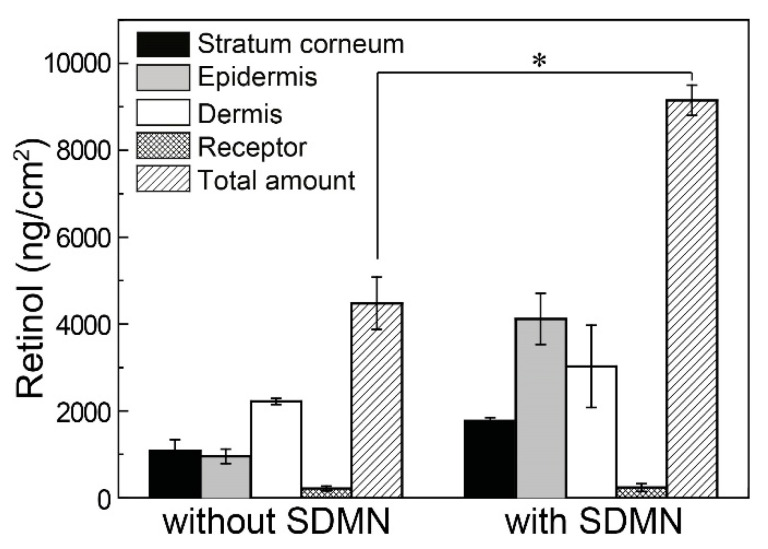
Retinol content in different skin compartments (SC, epidermis, dermis, and receptor) after 17 h of retinol nanocapsule application in the absence or presence of the SDMN patch. Mean ± SD, *n* = 2. * *p* < 0.05, statistically significant difference between the control group (without SDMN) and the SDMN-combined group.

Retinol was first encapsulated in a lipid matrix to facilitate chemical stabilization and water dispersion; the hydrodynamic diameter of the capsules was 349 nm (refer to Section 2). After the application of the SDMN patch, the retinol capsule-loaded aqueous solution was applied to the drug inlet hole for 17 h. As shown in Figure 6, the SDMN-combined group showed a permeation of 9.2 ± 0.3 μg/cm^2^, which was higher than that in the control group (4.5 ± 0.6 μg/cm^2^). In particular, when applied using the SDMN patch, the quantity of retinol delivered under the SC was clearly enhanced compared to that observed in the control group. It was reported that 1.4 μg of retinoic acid-loaded microneedle patch (area of 0.8 cm^2^) application induced epidermal cell proliferation and significantly accelerated the SC turnover [48]. Since retinol exhibited more than half the magnitude of the efficacy of retinoic acid at the same concentration [49], the level of retinol delivered by the SDMN patch has the potential to give the skin sufficient efficacy. In other words, the SDMN patch enables the delivery of small hydrophilic drugs as well as nanocapsulated lipophilic drugs with improved delivery efficiency and bioavailability.

## 4. Conclusions

TOBCNs owing to their excellent water affinity and the ability to retain their fiber structure in the aqueous phase, were successfully introduced into DMNs, and imparted a semi-dissolving characteristic. Using the structural characteristics of TOBCNs, a two-layer SDMN patch consisting of a soluble needle layer and an insoluble backing layer containing TOBCNs was fabricated in a one-step process, without the conventional two-step casting process. Upon the injection of the drug in the aqueous solution into the inlet hole in the backing layer, the TOBCNs served as a reservoir that quickly absorbed and continuously supplied the solution into the needle layer. The drug could be delivered through the micro-channels created after the needles dissolved in the aqueous solution and body fluids. In the in vitro skin permeation study, compared to the application of only the solution, the application of the SDMN patch facilitated the delivery of a high quantity of drugs without drug loading in the patch. These results are expected to be useful in the field of cosmetics and biomedical research in the future.

## Supplementary Materials

The following are available online at https://www.mdpi.com/2073-4360/12/9/1873/s1. Figure S1: SEM images showing the needle surface and the front surface of the SDMN; Figure S2: Compartmental distribution of fluorescein in different skin compartments after 3.5 h of application in the absence and presence of the SDMN patch.

## References

[1] Ma Y., Gill H.S. (2014). Coating solid dispersions on microneedles via a molten dip-coating method: Development and in vitro evaluation for transdermal delivery of a water-insoluble drug. J. Pharm. Sci..

[2] Vora L.K., Vavia P.R., Larrañeta E., Bell S.E.J., Donnelly R.F. (2018). Novel nanosuspension-based dissolving microneedle arrays for transdermal delivery of a hydrophobic drug. J. Interdiscip. Nanomed..

[3] Kim E., Erdos G., Huang S., Kenniston T.W., Balmert S.C., Carey C.D., Raj V.S., Epperly M.W., Klimstra W.B., Haagmans B.L. (2020). Microneedle array delivered recombinant coronavirus vaccines: Immunogenicity and rapid translational development. EBioMedicine.

[4] Fang J.-H., Liu C.-H., Hsu R.-S., Chen Y.-Y., Chiang W.-H., Wang H.-M.D., Hu S.-H. (2020). Transdermal Composite Microneedle Composed of Mesoporous Iron Oxide Nanoraspberry and PVA for Androgenetic Alopecia Treatment. Polymers.

[5] Guillot A.J., Cordeiro A.S., Donnelly R.F., Montesinos M.C., Garrigues T.M., Melero A. (2020). Microneedle-Based Delivery: An Overview of Current Applications and Trends. Pharmaceutics.

[6] Henry S., McAllister D.V., Allen M.G., Prausnitz M.R. (1998). Microfabricated microneedles: A novel approach to transdermal drug delivery. J. Pharm. Sci..

[7] Cormier M., Johnson B., Ameri M., Nyam K., Libiran L., Zhang D.D., Daddona P. (2004). Transdermal delivery of desmopressin using a coated microneedle array patch system. J. Control. Release.

[8] McAllister D.V., Allen M.G., Prausnitz M.R. (2000). Microfabricated microneedles for gene and drug delivery. Annu. Rev. Biomed. Eng..

[9] Okamura A.M., Simone C., O’Leary M.D. (2004). Force modeling for needle insertion into soft tissue. IEEE Trans. Biomed. Eng..

[10] Lee C., Eom Y.A., Yang H., Jang M., Jung S.U., Park Y.O., Lee S.E., Jung H. (2018). Skin barrier restoration and moisturization using horse oil-loaded dissolving microneedle patches. Ski. Pharmacol. Physiol..

[11] Lee S.G., Jeong J.H., Lee K.M., Jeong K.H., Yang H., Kim M., Jung H., Lee S., Choi Y.W. (2014). Nanostructured lipid carrier-loaded hyaluronic acid microneedles for controlled dermal delivery of a lipophilic molecule. Int. J. Nanomed..

[12] Limcharoen B., Toprangkobsin P., Kröger M., Darvin M.E., Sansureerungsikul T., Rutwaree T., Wanichwecharungruang S., Banlunara W., Lademann J., Patzelt A. (2020). Microneedle-Facilitated Intradermal Proretinal Nanoparticle Delivery. Nanomaterials.

[13] Kim S., Dangol M., Kang G., Lahiji S.F., Yang H., Jang M., Ma Y., Li C., Lee S.G., Kim C.H. (2017). Enhanced transdermal delivery by combined application of dissolving microneedle patch on serum-treated skin. Mol. Pharm..

[14] Kang G., Kim S., Yang H., Jang M., Chiang L., Baek J.H., Ryu J.H., Choi G.W., Jung H. (2019). Combinatorial application of dissolving microneedle patch and cream for improvement of skin wrinkles, dermal density, elasticity, and hydration. J. Cosmet. Dermatol..

[15] Yang H., Kim S., Jang M., Kim H., Lee S., Kim Y., Eom Y.A., Kang G., Chiang L., Baek J.H. (2019). Two-phase delivery using a horse oil and adenosine-loaded dissolving microneedle patch for skin barrier restoration, moisturization, and wrinkle improvement. J. Cosmet. Dermatol..

[16] Ragauskas A.J., Williams C.K., Davison B.H., Britovsek G., Cairney J., Eckert C.A., Frederick W.J., Hallett J.P., Leak D.J., Liotta C.L. (2006). The path forward for biofuels and biomaterials. Science.

[17] Abeer M.M., Mohd Amin M.C.I., Martin C. (2014). A review of bacterial cellulose-based drug delivery systems: Their biochemistry, current approaches and future prospects. J. Pharm. Pharmacol..

[18] Plackett D., Letchford K., Jackson J., Burt H. (2014). A review of nanocellulose as a novel vehicle for drug delivery. Nord. Pulp Pap. Res. J..

[19] Abdul Khalil H.P.S., Adnan A.S., Yahya E.B., Olaiya N.G., Safrida S., Hossain M.S., Balakrishnan V., Gopakumar D.A., Abdullah C.K., Oyekanmi A.A. (2020). A Review on Plant Cellulose Nanofibre-Based Aerogels for Biomedical Applications. Polymers.

[20] Amnuaikit T., Chusuit T., Raknam P., Boonme P. (2011). Effects of a cellulose mask synthesized by a bacterium on facial skin characteristics and user satisfaction. Med. Devices (Auckl).

[21] Pacheco G., de Mello C.V., Chiari-Andréo B.G., Isaac V.L.B., Ribeiro S.J.L., Pecoraro É., Trovatti E. (2018). Bacterial cellulose skin masks-Properties and sensory tests. J. Cosmet. Dermatol..

[22] Chen W., Yu H., Lee S.-Y., Wei T., Li J., Fan Z. (2018). Nanocellulose: A promising nanomaterial for advanced electrochemical energy storage. Chem. Soc. Rev..

[23] Lee B.-M., Lee C., Lahiji S.F., Jung U.-W., Chung G., Jung H. (2020). Dissolving Microneedles for Rapid and Painless Local Anesthesia. Pharmaceutics.

[24] Tahara N., Tabuchi M., Watanabe K., Yano H., MoRinaga Y., Yoshinaga F. (1997). Degree of polymerization of cellulose from Acetobacter xylinum BPR2001 decreased by cellulase produced by the strain. Biosci. Biotechnol. Biochem..

[25] Naritomi T., Kouda T., Yano H., Yoshinaga F. (1998). Effect of lactate on bacterial cellulose production from fructose in continuous culture. J. Ferment. Bioeng..

[26] Lee J.W., Deng F., Yeomans W.G., Allen A.L., Gross R.A., Kaplan D.L. (2001). Direct incorporation of glucosamine and N-acetylglucosamine into exopolymers by Gluconacetobacter xylinus (=Acetobacter xylinum) ATCC 10245: Production of chitosan-cellulose and chitin-cellulose exopolymers. Appl. Environ. Microbiol..

[27] Svensson A., Nicklasson E., Harrah T., Panilaitis B., Kaplan D.L., Brittberg M., Gatenholm P. (2005). Bacterial cellulose as a potential scaffold for tissue engineering of cartilage. Biomaterials.

[28] Pasaribu K.M., Gea S., Ilyas S., Tamrin T., Sarumaha A.A., Sembiring A., Radecka I. (2020). Fabrication and in-Vivo Study of Micro-Colloidal Zanthoxylum acanthopodium-Loaded Bacterial Cellulose as a Burn Wound Dressing. Polymers.

[29] Ashour R.M., Abdel-Magied A.F., Wu Q., Olsson R.T., Forsberg K. (2020). Green Synthesis of Metal-Organic Framework Bacterial Cellulose Nanocomposites for Separation Applications. Polymers.

[30] Jun S.-H., Lee S.-H., Kim S., Park S.-G., Lee C.-K., Kang N.-K. (2017). Physical properties of TEMPO-oxidized bacterial cellulose nanofibers on the skin surface. Cellulose.

[31] Jun S.-H., Park S.-G., Kang N.-G. (2019). One-pot method of synthesizing TEMPO-oxidized bacterial cellulose nanofibers using immobilized TEMPO for skincare applications. Polymers.

[32] McAllister D.V., Wang P.M., Davis S.P., Park J.-H., Canatella P.J., Allen M.G., Prausnitz M.R. (2003). Microfabricated needles for transdermal delivery of macromolecules and nanoparticles: Fabrication methods and transport studies. Proc. Natl. Acad. Sci. USA.

[33] Chen K.Y., Ren L., Chen Z.P., Pan C.F., Zhou W., Jiang L.L. (2016). Fabrication of micro-needle electrodes for bio-signal recording by a magnetization-induced self-assembly method. Sensors.

[34] Gao Y., Hou M., Yang R., Zhang L., Xu Z., Kang Y., Xue P. (2019). Highly porous silk fibroin scaffold packed in PEGDA/sucrose microneedles for controllable transdermal drug delivery. Biomacromolecules.

[35] Jeon E.Y., Lee J., Kim B.J., Joo K., Kim K.H., Lim G., Cha H.J. (2019). Bio-inspired swellable hydrogel-forming double-layered adhesive microneedle protein patch for regenerative internal/external surgical closure. Biomaterials.

[36] Cho W.K., Ankrum J.A., Guo D., Chester S.A., Yang S.Y., Kashyap A., Campbell G.A., Wood R.J., Rijal R.K., Karnik R. (2012). Microstructured barbs on the North American porcupine quill enable easy tissue penetration and difficult removal. Proc. Natl. Acad. Sci. USA.

[37] Khanna P., Luongo K., Strom J.A., Bhansali S. (2010). Sharpening of hollow silicon microneedles to reduce skin penetration force. J. Micromech. Microeng..

[38] Larrañeta E., Moore J., Vicente-Pérez E.M., González-Vázquez P., Lutton R., Woolfson A.D., Donnelly R.F. (2014). A proposed model membrane and test method for microneedle insertion studies. Int. J. Pharm..

[39] Vos P.J., Kuijt N., Kaya M., Rol S., van der Maaden K. (2020). Nanoporous microneedle arrays seamlessly connected to a drug reservoir for tunable transdermal delivery of memantine. Eur. J. Pharm. Sci..

[40] Donnelly R.F., Singh T.R.R., Garland M.J., Migalska K., Majithiya R., McCrudden C.M., Kole P.L., Mahmood T.M.T., McCarthy H.O., Woolfson A.D. (2012). Hydrogel-forming microneedle arrays for enhanced transdermal drug delivery. Adv. Funct. Mater..

[41] Donnelly R.F., Singh T.R.R., Alkilani A.Z., McCrudden M.T.C., O’Neill S., O’Mahony C., Armstrong K., McLoone N., Kole P., Woolfson A.D. (2013). Hydrogel-forming microneedle arrays exhibit antimicrobial properties: Potential for enhanced patient safety. Int. J. Pharm..

[42] Kearney M.-C., McKenna P.E., Quinn H.L., Courtenay A.J., Larrañeta E., Donnelly R.F. (2019). Design and Development of Liquid Drug Reservoirs for Microneedle Delivery of Poorly Soluble Drug Molecules. Pharmaceutics.

[43] Bellemère G., Stamatas G.N., Bruère V., Bertin C., Issachar N., Oddos T. (2009). Antiaging Action of Retinol: From Molecular to Clinical. Ski. Pharmacol. Physiol..

[44] Lee M.-H., Oh S.-G., Moon S.-K., Bae S.-Y. (2001). Preparation of silica particles encapsulating retinol using O/W/O multiple emulsions. J. Colloid Interface Sci..

[45] Müller R.H., Radtke M., Wissing S.A. (2002). Nanostructured lipid matrices for improved microencapsulation of drugs. Int. J. Pharm..

[46] Jee J.-P., Lim S.-J., Park J.-S., Kim C.-K. (2006). Stabilization of all-trans retinol by loading lipophilic antioxidants in solid lipid nanoparticles. Eur. J. Pharm. Biopharm..

[47] Eskandar N.G., Simovic S., Prestidge C.A. (2009). Chemical stability and phase distribution of all-trans-retinol in nanoparticle-coated emulsions. Int. J. Pharm..

[48] Hiraishi Y., Hirobe S., Iioka H., Quan Y.S., Kamiyama F., Asada H., Okada N., Nakagawa S. (2013). Development of a novel therapeutic approach using a retinoic acid-loaded microneedle patch for seborrheic keratosis treatment and safety study in humans. J. Control. Release.

[49] Kong R., Cui Y., Fisher G.J., Wang X., Chen Y., Schneider L.M., Majmudar G. (2016). A comparative study of the effects of retinol and retinoic acid on histological, molecular, and clinical properties of human skin. J. Cosmet. Dermatol..

