# Supplementary material for: A Semi-Dissolving Microneedle Patch Incorporating TEMPO-Oxidized Bacterial Cellulose Nanofibers for Enhanced Transdermal Delivery
Source: Polymers (Basel). 2020 Aug 20;12(9):1873. doi: 10.3390/polym12091873 (PMC7564169; doi:10.3390/polym12091873)
Supplement: Supplementary file 1 [file polymers-12-01873-s001.pdf]

Supporting Information

A Semi-Dissolving Microneedle Patch Incorporating TEMPO-Oxidized Bacterial Cellulose Nanofibers for Enhanced Transdermal Delivery

Ji Eun Song †, Seung-Hyun Jun †, Sun-Gyoo Park and Nae-Gyu Kang *

LG Household & Health Care R&D Center, Seoul 07795, Korea; sos6934@lghnh.com (J.E.S.); junsh@lghnh.com (S.-H.J.); skparke@lghnh.com (S.-G.P.)

* Correspondence: ngkang@lghnh.com; Tel.: +82-2-6980-1533

† These two authors equally contribute to this work.

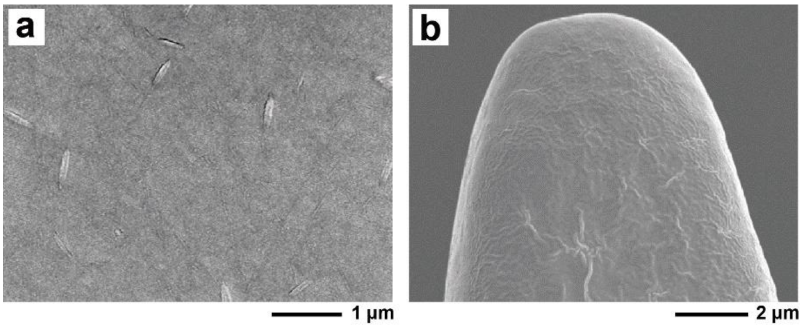

Figure S1. SEM images showing (**a**) the front side (needles were distributed) of the backing layer and (**b**) the needle surface of the SDMN patch.

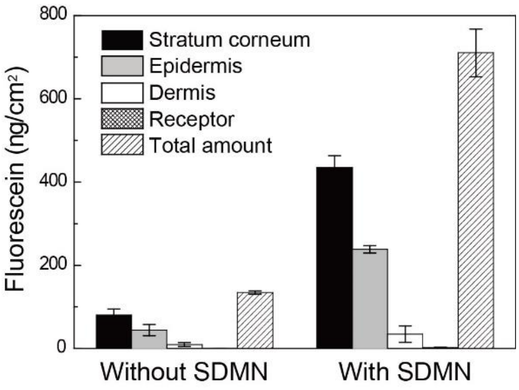

Figure S2. Compartmental distribution of fluorescein in the SC, epidermis, dermis and receptor medium after 3.5 h of application in the absence and presence of the SDMN patch.
